# Pharmacology of the Phosphate Binder, Lanthanum Carbonate

**DOI:** 10.3109/0886022X.2011.552821

**Published:** 2011-02-18

**Authors:** Stephen JP Damment

**Affiliations:** Shire Pharmaceuticals, Hampshire International Business Park, Chineham, Basingstoke, UK

**Keywords:** chronic kidney disease, kidney diseases, hyperphosphatemia, lanthanum carbonate, phosphate binder

## Abstract

Studies were conducted to compare the phosphate-binding efficacy of lanthanum carbonate directly with other clinically used phosphate binders and to evaluate any potential adverse pharmacology. To examine the phosphate-binding efficacy, rats with normal renal function and chronic renal failure received lanthanum carbonate, aluminum hydroxide, calcium carbonate, or sevelamer hydrochloride in several experimental models. Lanthanum carbonate and aluminum hydroxide markedly increased excretion of [^32^P]-phosphate in feces and reduced excretion in urine in rats with normal renal function (*p* < 0.05), indicating good dietary phosphate-binding efficacy. In rats with chronic renal failure, lanthanum carbonate and aluminum hydroxide reduced urinary phosphate excretion to a greater degree and more rapidly than calcium carbonate, which in turn was more effective than sevelamer hydrochloride. The potential to induce adverse pharmacological effects was assessed systematically in mice, rats, and dogs with normal renal function using standard in vivo models. There was no evidence of any adverse secondary pharmacological effects of lanthanum carbonate on the central nervous, cardiovascular, respiratory, or gastrointestinal systems. These studies indicate that lanthanum carbonate is the more potent of the currently available dietary phosphate binders. No adverse secondary pharmacological actions were observed in vivo in a systematic evaluation at high doses.

## INTRODUCTION

Hyperphosphatemia is associated with significant morbidity and mortality in patients with chronic kidney disease,^1^ and despite treatment, more than half of patients on dialysis have serum phosphorus levels above Kidney Disease Outcomes Quality Initiative recommendations (3.5-5.5 mg/dL).^2^ Analysis of recent data suggests that sustained control of serum phosphorus levels within the range 3.5-5.5 mg/dL can reduce mortality risk in patients on dialysis,^3^ indicating a critical need to manage serum phosphorus levels effectively in these patients.

Restriction of dietary phosphate intake to 800-1000 mg/day is recommended in patients on dialysis whose serum phosphorus levels exceed 5.5 mg/dL.^4^ Unfortunately, low-phosphate diets can be unpalatable,^5^ and the risk of protein malnutrition may outweigh any benefit of controlling serum phosphorus levels. The administration of dietary phosphate binders is therefore necessary in most patients on dialysis to effectively manage their hyperphosphatemia.

Use of aluminum-based phosphate binders was largely abandoned in the 1980s because of concerns over accumulation and major toxicities including dialysis encephalopathy and osteomalacia.^7^ Calcium-based agents replaced aluminum but as shown by the theoretical, in vitro, and healthy volunteer balance studies of Sheikh et al., not all calcium-based agents are as effective as aluminum.^8^ The requirement for high daily doses of calcium-based binders may increase the risk of hypercalcemia, adynamic bone disease, and vascular calcification.^7^ The need for effective treatments with better safety profiles led to the introduction of the new generation of non-calcium-based phosphate binders, initially sevelamer hydrochloride^9^ and then lanthanum carbonate.^10^

Large comparative clinical studies of lanthanum carbonate have generally used a variable dose treat-to-target design that does not allow direct comparison of phosphate-binding potency with other binders;^11^,^12^ studies have concentrated on differences in the surrogate measure of tablet burden.^13^ However, in vitro experiments and short-term clinical studies have investigated the comparative efficacy of lanthanum carbonate and sevelamer hydrochloride.^14^,^15^ To our knowledge, no published studies have directly compared the in vivo efficacy of lanthanum carbonate with other available phosphate binders in preclinical models.

Similarly, although the calcium-like pharmacology of lanthanum in in vitro systems is well known, the potential of lanthanum carbonate to induce adverse effects through calcium-like actions has not been evaluated comprehensively in standard in vivo safety-pharmacology studies.

The aims of the investigations described here were to directly compare the phosphate-binding efficacy of lanthanum carbonate with other clinically used phosphate binders in vivo and to systematically evaluate any potential of lanthanum to induce adverse pharmacological effects in standard in vivo models.

## MATERIALS AND METHODS

Lanthanum carbonate was supplied by Catalytica Pharmaceuticals Inc. (Greenville, NC, USA), Johnson Matthey (London, UK), Quintiles Scotland Ltd. (Edinburgh, UK), or Shire Pharmaceutical Development Ltd. (Basingstoke, UK). Sevelamer hydrochloride was supplied by Genzyme BV (Naarden, the Netherlands). Calcium carbonate and aluminum hydroxide were supplied by Sigma Chemical Co. (St. Louis, MO, USA) or Sigma-Aldrich (Poole, UK).

### Primary Pharmacology

Rats were supplied by Charles River Laboratories Ltd. (Margate, UK), Iffa Credo (Brussels, Belgium), or Harlan Sprague Dawley Inc. (Indianapolis, IN, USA). Animals were housed individually or in groups and transferred to individual metabolic cages when required. Protocols adhered to the ‘Principles of Laboratory Animal Care’ (NIH publication #85-23, revised in 1985), were approved, and complied with national laws and guidelines.

Lanthanum carbonate, aluminum hydroxide, and calcium carbonate were administered by oral gavage in 0.5-2% (w/v) carboxymethyl cellulose (Sigma-Aldrich Inc., Milwaukee, WI, USA). Sevelamer hydrochloride was administered in water. All phosphate binders were administered in a volume of 10-20 mL/kg body weight per day.

In experiments requiring a state of chronic renal failure, anesthetized rats underwent 5/6th nephrectomy, performed in two stages by ligation of two of the three branches of the left kidney artery, 1 week before complete removal of the right kidney. Animals were allowed to stabilize postsurgery, before treatments were initiated.

#### Effect of lanthanum carbonate and aluminum hydroxide on excretion of [^32^P]-phosphate in rats with normal renal function

Male Sprague Dawley rats (Crl: CD® BR, *n* = 5 per group; ∼8 weeks old; weight range = 100-200 g) received vehicle, lanthanum carbonate (1000 mg/kg/day), or aluminum hydroxide (1000 mg/kg/day) for 12 days. On day 7, a single dose of radiolabeled [^32^P]-phosphate (11 μCi) was administered to each animal by oral gavage 30 min following dosing with phosphate binder. Urine (including cage wash) and feces were collected continuously for 144 h post-[^32^P]-phosphate dose. Excretion samples were stored below -20°C before analysis. Fecal samples were solubilized in Soluene®-350 (Canberra Packard, Reading, UK) before mixing with Hionic Fluor scintillant (Canberra Packard), whereas urine samples were mixed with Ultima Gold scintillant (Canberra Packard). Radioactive content was determined in triplet aliquots, by liquid scintillation counting (Canberra Packard). Data are presented as mean cumulative percentage of administered [^32^P]-phosphate dose ± standard deviation (SD). Data were analyzed using a Kruskal-Wallis test followed by a pairwise Mann-Whitney test.

#### Effect of lanthanum carbonate on calcium and phosphate metabolism in rats with normal renal function or chronic renal failure

Male rats with normal renal function [Crl: CD®(SD)BR strain VAF plus, *n* = 10 per group; <6 weeks old; weight range = 141-169 g] were dosed daily for 13 weeks with vehicle or lanthanum carbonate (200, 600, or 2000 mg/kg/day). During week 13, blood samples were taken by retro-orbital sinus puncture from animals anesthetized under halothane after an overnight fast. Serum calcium and phosphorus levels were measured in samples prepared in lithium-heparin anticoagulant. Parathyroid hormone (PTH) levels were determined in plasma samples isolated by blood coagulation at room temperature followed by centrifugation and subsequent plasma precipitation at less than -18°C. Urinary phosphate concentrations were also determined in samples collected overnight. Data are presented as mean ± SD. Data were analyzed by analysis of variance, followed by pairwise comparison of all treatment groups versus the vehicle-treated control group using the Williams test. For the comparison of the 2000 mg/kg/day group versus the control group, a two-sided test was performed (significance at 5% level). If this comparison was not significant then testing was stopped, otherwise the process continued (using a one-sided test) with the lower dose groups until a nonsignificant comparison was encountered.

In a second study, chronic renal failure was induced by 5/6th nephrectomy. Male Wistar rats (*n* = 10 per group) were treated with vehicle or lanthanum carbonate (100, 500, 1000, or 2000 mg/kg/day) for 12 weeks. Blood and urine biochemistry were examined. Detailed methodology is described in Behets et al.^16^ Data are presented as mean ± SD. For each time point and variable, a Kruskal-Wallis test was performed to test for differences between groups, followed by a Mann-Whitney U-test with Bonferroni correction when significant differences between groups were found. Differences between groups were considered significant at *p* < 0.05.

#### Relative urinary phosphate-lowering effect of lanthanum carbonate, aluminum hydroxide, calcium carbonate, and sevelamer hydrochloride in rats with chronic renal failure

Male Sprague Dawley rats (aged ∼12 weeks; weight range = 280–300 g) underwent 5/6th nephrectomy. After a 2-week recovery period, animals were dosed daily for 6 weeks with 1000 mg/kg/day of lanthanum carbonate, aluminum hydroxide, calcium carbonate, sevelamer hydrochloride, or vehicle (*n* = 10 for each treatment group). Twenty-four-hour urine samples were collected before surgery and after 0, 2, 4, and 6 weeks of treatment. Urine samples were centrifuged to remove sediment and aliquots were analyzed colorimet-rically for phosphate. Data are reported as mean ± SD. Data were analyzed using a one-way analysis of variance. The comparison between treatment groups was made using Dunnett's procedure with the vehicle-treated Control group as a reference.

### Safety Pharmacology

The safety pharmacology of lanthanum carbonate was investigated thoroughly in mice, rats, and dogs, with standard methods applied to identify adverse pharmacological effects on the cardiovascular and respiratory systems, central nervous system (CNS), and gastrointestinal (GI) tract. The studies are listed in Table 1, and the methods employed can be found in publications by the International Conference on Harmonisation of Technical Requirements for Registration of Pharmaceuticals for Human Use^17^ and Vogel et al.^18^ All doses of lanthanum carbonate refer to the salt and not the cation.

**Table 1:** Assessment of the safety pharmacology of lanthanum carbonate.

Species	Study	Objective	Dose of lanthanum carbonate	Main findings
Cardiovascular system				
Dog	SPD/64/PH	Effects on cardiovascular function (anesthetized model)	200, 600, or 2000 mg/kg (single dose, intraduodenal)	No effects of treatment on blood pressure (femoral artery), heart rate, left ventricular pressure, ECG waveform, or blood flow.
Dog	SPD0104	Effects on cardiovascular function (4-week treatment)	0.003, 0.05, or 1 mg/kg/day (28 days, intravenous chloride salt)	No treatment-related effects on ECG waveform. Doses gave plasma lanthanum *C*_max_ values up to 20,000× those in humans (1 ng/mL).
Respiratory system				
Dog	SPD/64/PH	Effects on respiratory function (anesthetized model)	200, 600, or 2000 mg/kg (single dose, intraduodenal)	No effects on respiration rate, tidal volume, or minute volume.
Central nervous system				
Rat	SPD/41/96	Effects on neurobehavior	2000 mg/kg (single, oral gavage)	Behavior unaffected and no overt signs of toxicity.
Mouse	SPD/42/96	Effects on neurobehavior	500, 1000, or 2000 mg/kg (single, oral gavage)	Behavior unaffected (with the exception of piloerection in two males, 4 h after administration of 2000 mg/kg) and there were no signs of toxicity.
Mouse	SPD/62/PH	Effects on body temperature and behavior	200, 500, or 1000 mg/kg (single, oral gavage)	No effects on body temperature or pharmacological activity detectable by the Irwin test (all doses).
Mouse	SPD/63/PH	Effects on spontaneous motor activity	200, 500, or 1000 mg/kg (single, oral gavage)	Line crossing values comparable with controls; no effect on spontaneous motor activity (all doses).
Mouse	SRU 003/992850	Potential to induce proconvulsant activity	500, 1000, or 2000 mg/kg (single, oral gavage)	No statistically significant proconvulsant activity in either the metrazol- or electroshock-induced tests, compared with the vehicle-treated control group.
Mouse	SRU 005/992851	Potential to induce anticonvulsant activity	500, 1000, or 2000 mg/kg (single, oral gavage)	No significant anticonvulsant activity in either the minimal metrazol or supramaximal electroshock test (all doses).
Dog	SPD/66/TK	Effects on neurobehavior, chronic treatment	200, 600, or 2000 mg/kg/day (52-week treatment, oral capsule)	Assessment included anal sphincter tone, gait, patella reflex, panniculus reflex, pupillary response, ocular cephalic reflex, palpebral reflex, proprioception, righting reflex, withdrawal reflex. No treatment-related findings in the neurotoxicity assessment (all doses).
Mouse	SPD/88/C	Effects on neurobehavior, chronic treatment	100 or 1500 mg/kg/day (62-week treatment, oral gavage)	No behavioral, autonomic, or neurologic effects (Irwin test, all doses).
Gastrointestinal tract				
Rat	SPD/47/PH	Effects on charcoal meal transit in the small intestine	200, 500, or 1000 mg/kg (single, oral gavage)	200, 500, or 1000 mg/kg (single, oral gavage)
Rat	SPD/49/PH	Effects on urinary and fecal output	200, 500, or 1000 mg/kg (single, oral gavage)	No diarrhea or abnormal fecal output suggesting no disturbance of intestinal function.
Rat	SPD/48/PH	Effects on gastric acid secretion	200, 500, or 1000 mg/kg (single, oral gavage)	No effect on volume of gastric acid secretion. Reduced acidity of gastric contents by neutralization at 1000 mg/kg.
Rat	SPD/50/PH	Potential to induce lesions in the stomach 200, 500, or 1000 mg/kg (single, oral gavage)	No gastric damage.	
Rat	SPD/51/PH	Effects on aspirin-induced gastric damage	200, 500, or 1000 mg/kg (single, oral gavage)	No exacerbation of aspirin-induced gastric damage.

Note: ECG, electrocardiogram.

## RESULTS

### Primary Pharmacology

#### Effect of lanthanum carbonate and aluminum hydroxide treatment on excretion of f P]-phosphate in rats with normal renal function

**Figure 1:**
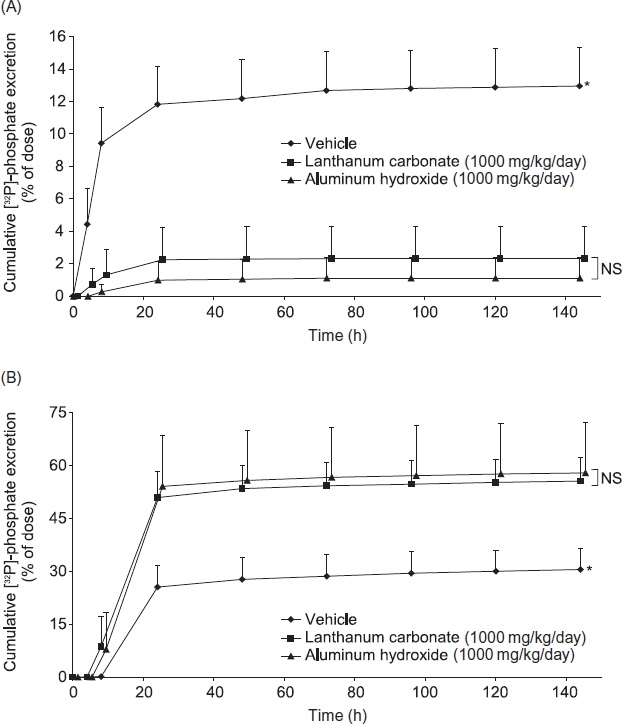
The effects of lanthanum carbonate and aluminum hydroxide on urinary (A) and fecal (B) excretion of [^32^P]-phosphate in rats with normal renal function. Rats (*n* = 5 per group) were treated with vehicle, lanthanum carbonate, or aluminum hydroxide by oral gavage for 12 days. On day 7, animals received a single oral dose of 11 μCi [^32^P]-phosphate. Urine and feces were then collected continuously for 144 h and sample radioactivity was determined. Data are mean ± SD. Notes: *Denotes *p* < 0.05 versus lanthanum carbonate and aluminum hydroxide; NS, not significant.

The effects of oral administration of lanthanum carbonate and aluminum hydroxide on mean urinary and fecal excretion of [^32^P]-phosphate are shown in Figure 1. Following the oral administration of [^32^P]-phosphate in vehicle-treated animals, 13.0 ± 2.4% of the administered dose was recovered in urine within 144 h. Treatment of rats with lanthanum carbonate (1000 mg/kg/ day) for 6 days before administration of [^32^P]-phosphate resulted in a statistically significant reduction (82.2%) in recovered radioactivity (i.e., 2.3 ± 2.0% of the administered dose) compared with vehicle-treated controls (*p* < 0.05) (Figure 1A). Lanthanum carbonate treatment also resulted in a statistically significant increase (82.1%) in fecal [^32^P]-phosphate excretion compared with vehicle-treated controls (55.6 ± 6.6% vs. 30.5 ± 5.9% of the administered dose, respectively, *p* < 0.05) (Figure 1B). At the same dose (1000 mg/kg/day), treatment with aluminum hydroxide also resulted in a marked reduction of mean urinary phosphate excretion (91.4%, i.e., 1.1 ± 1.3% in the administered dose, *p* < 0.05), with an increase in fecal [^32^P]-phosphate excretion (89.7%, i.e., 57.9 ± 14.2% of the administered dose, *p* < 0.05). There were no significant differences in the reduction in urinary [^32^P]-phosphate excretion and the increase in fecal [^32^P]-phosphate excretion between lanthanum carbonate and aluminum hydroxide groups.

#### Effect of lanthanum carbonate on calcium and phosphate metabolism in rats with normal renal function or chronic renal failure

The effect of lanthanum carbonate treatment on serum and urine biochemistry was investigated in rats with normal renal function during a 13-week study. The same parameters were then assessed in a 12-week study in rats with 5/6th nephrectomy-induced chronic renal failure.

**Figure 2:**
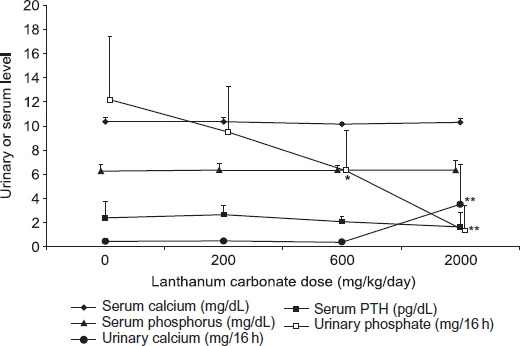
Effect of lanthanum carbonate on phosphate, calcium, and parathyroid hormone (PTH) in rats with normal renal function. Rats (*n* = 10 per group) were dosed daily by oral gavage for 13 weeks with vehicle or lanthanum carbonate. Blood and urine samples were then collected during fasting conditions and phosphorus, calcium, and PTH levels were determined. Data are presented as mean ± SD. Note: * and ** Denote *p* < 0.01 and *p* < 0.001, respectively, versus vehicle-treated control group.

In rats with normal renal function, lanthanum carbonate treatment (200-2000 mg/kg/day) resulted in a dose-dependent reduction in urinary phosphate excretion. Mean urinary phosphate excretion was significantly lower in the 600 mg/kg/day and the 2000 mg/kg/ day lanthanum carbonate groups (6.3 ± 3.3 and 1.4 ± 2.0 mg/16 h, *p* < 0.01 and *p* < 0.001, respectively) compared with vehicle-treated controls (12.1 ± 5.3 mg/ 16 h, Figure 2). No significant changes in serum phosphorus, PTH, or calcium levels were observed in rats with increasing doses of lanthanum carbonate. The urinary calcium concentration was also unaffected by lanthanum carbonate treatment, except for a small but significant increase at the highest dose (2000 mg/ kg/day, *p* < 0.001 compared with vehicle-treated controls).

**Figure 3:**
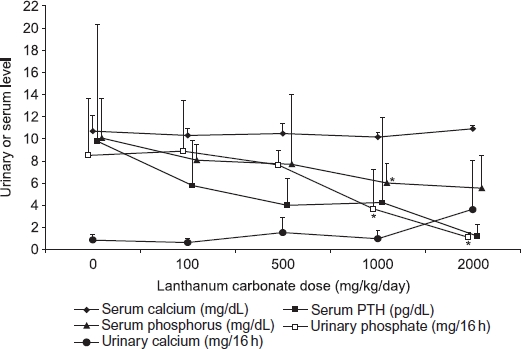
Effect of lanthanum carbonate on phosphate, calcium, and parathyroid hormone (PTH) in rats with chronic renal failure. Rats (*n* = 10 per group) underwent 5/6th nephrectomy and were administered vehicle or lanthanum carbonate by oral gavage for 12 weeks. Blood and urine samples were then collected during fasting conditions and levels of phosphorus, calcium, and PTH were determined. Data are presented as mean ± SD. Note: *Denotes *p* < 0.05 versus vehicle-treated control group.^16^

In rats with 5/6th nephrectomy-induced chronic renal failure (Figure 3), the levels of serum phosphorus and PTH observed in vehcile-treated control animals after 12 weeks of treatment were higher than those seen in animals with normal renal function treated for 13 weeks in the previous study, as expected. Lanthanum carbonate treatment resulted in dose-dependent but not significant reductions in mean serum phosphorus and PTH levels (5.6 ± 3.0 mg/dL and 1.2 ± 1.1 pg/dL, respectively, at 2000 mg/kg/day), restoring these parameters to the levels in vehicle-treated rats with normal renal fun ction (6.3 ± 0.5 mg/dL and 2.4 ± 1.3 pg/dL, respectively). Lanthanum carbonate treatment caused a dose-dependent reduction in urinary phosphate excretion, similar to the effect observed in rats with normal renal function. Mean urinary phosphate concentrations were significantly lower in rats treated with 2000 mg/kg/day lanthanum carbonate (1.1 ± 0.4 mg/24 h, *p* < 0.05) compared with vehicle-treated controls (8.5 ± 5.1 mg/24 h; Figure 3). In rats with chronic renal failure, treatment with the highest dose of lanthanum carbonate (2000 mg/kg/day) resulted in a nonsignificant increase in urinary calcium excretion. This effect was similar to that observed in rats with normal renal function.

#### Relative urinary phosphate-lowering effect of lanthanum carbonate, aluminum hydroxide, calcium carbonate, and sevelamer hydrochloride in rats with chronic renal failure

**Figure 4:**
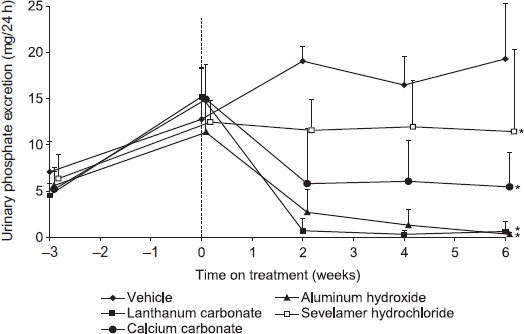
Relative urinary phosphate-lowering effect of lanthanum carbonate, aluminum hydroxide, calcium carbonate, and sevelamer hydrochloride in rats with chronic renal failure. Rats (*n* = 10) underwent 5/6th nephrectomy. Following stabilization postsurgery, animals were treated with vehicle or 1000 mg/kg/day of lanthanum carbonate, aluminum hydroxide, calcium carbonate, or sevelamer hydrochloride by oral gavage for 6 weeks. At weeks —3, 0, 2, 4, and 6, 24-h urine samples were collected and analyzed for phosphate levels. Data are presented as mean ± SD. Source: Adapted from Hutchison.^7^ Note: *Denotes *p* < 0.01 for least-squares (LS) mean change from baseline versus vehicle-treated control group.

Before surgical induction of chronic renal failure by 5/6th nephrectomy, mean urinary phosphate excretion was 7.1 ± 3.3 mg/24 h in control animals. This increased to 12.8 ± 5.6 mg/24 h at 3 weeks postsurgery and to 19.3 ± 6.0 mg/24 h by the end of the study (week 6). At a dose of 1000 mg/kg/day, all of the phosphate binders prevented the increase in urinary phosphate excretion observed in vehicle-treated animals (for all groups, *p* < 0.01 for least-squares mean change from baseline, compared with the vehicle-treated control group) (Figure 4). Aluminum hydroxide and lanthanum carbonate rapidly reduced mean urinary phosphate excretion and sustained the reduction to the end of the 6-week treatment period (98.3% and 96.7% reductions, respectively, compared with vehicle-treated controls at week 6). Mean urinary phosphate excretion was reduced to a lesser degree by calcium carbonate and sevelamer hydrochloride (71.7% and 40.7% respectively, compared with vehicle-treated controls at week 6).

### Safety Pharmacology

No adverse cardiovascular, respiratory, CNS, or GI pharmacology was identified in the International Conference on Harmonisation of Technical Requirements for Registration of Pharmaceuticals for Human Use^17^ core battery of safety-pharmacology studies (Table 1). The only pharmacological effect of note was an increase in gastric pH, observed in rats treated orally with lanthanum carbonate.

## DISCUSSION

### Primary Pharmacology

In normal rats, lanthanum carbonate reduced the absorption of orally administered [^32^P]-phosphate, as demonstrated by increased fecal excretion and reduced urinary excretion of radiolabel compared with vehicle-treated controls. Aluminum hydroxide, which is recognized as the most potent phosphate binder in clinical use,^7^ produced similar changes at the same dose. In rodent chronic renal failure models, the kidneys retain partial function and, under conditions of reduced phosphate availability, reabsorb phosphate from the urine to maintain serum concentrations.^16^ Thus, urinary phosphate excretion is a sensitive marker of phosphate-binder efficacy in these models; the greater the amount of phosphate bound and trapped in the intestinal lumen, the greater the amount reabsorbed in the kidney, and the smaller the amount excreted in urine. Data from uremic rats suggest that lanthanum carbonate and aluminum hydroxide are the most efficacious binders. Trends suggested more rapid and larger reductions in urinary phosphate excretion compared with equivalent gravimetric doses of sevelamer hydrochloride or calcium carbonate over a 6-week treatment period.

Serum phosphorus and PTH concentrations were not altered in normal rats dosed with lanthanum carbonate over a 3-month period, as expected, because homeostatic mechanisms for regulating mineral metabolism are intact. Increased renal resorption of phosphate and upregulation of 1,25-dihydroxyvitamin D synthesis were presumably adequate to maintain serum phosphorus concentrations within the normal range without the need to effect changes in PTH secretion and bone turnover.

In 5/6th nephrectomized rats, phosphate metabolism was disrupted and the effects of lanthanum carbonate treatment were distinct from those observed in normal rats. Serum phosphorus and PTH concentrations were elevated in the uremic controls compared with normal controls, as residual kidney function was inadequate to maintain serum phosphorus in the normal range. Treatment with lanthanum carbonate resulted in dose-related reductions in both of these parameters, towards the levels observed in animals with normal renal function. Similar effects have been observed with sevelamer hydrochloride treatment in rats with various models of chronic renal failure.^19^,^20^

Both normal and uremic rats exhibited a small increase in urine calcium excretion at high doses of lanthanum carbonate, without any significant changes in serum calcium concentration. This has been reported with other phosphate binders.^19^,^20^ Phosphate in the intestine acts as a natural calcium binder, and therefore the use of phosphate binders can lead to an increase in the amount of dietary calcium available for absorption.^21^ It is possible that the elevated urine calcium excretion is a consequence of this phenomenon. Alternatively, as lanthanum carbonate administration reduced urinary phosphate excretion almost to zero at 2000 mg/kg/day, rats may have been in negative balance at this dose due to over-efficient binding of phosphate in the intestine. Under these circumstances, phosphorus (and hence calcium) may have been mobilized from bone to maintain serum phosphorus concentrations in the physiologic range.

### Safety Pharmacology

In vitro, lanthanum ions have been widely shown to have both calcium agonist and antagonist actions, depending on the proteins or cell systems being investigated.^22^ The extent to which these effects are relevant to clinical use of lanthanum carbonate is contentious due to poor GI absorption and extensive protein binding,^23^ resulting in very low systemic levels of free lanthanum. The in vitro effects of lanthanum may be explained by its ability to substitute for calcium at binding sites on extracellular or cell-membrane-bound proteins.^22^ Intracellular processes are unaffected as lanthanum appears to be unable to enter healthy cells except by pinocytosis or phagocytosis.^22^

In our systematic evaluation of safety pharmacology, no adverse effects were observed on cardiovascular and respiratory systems in anesthetized instrumented dogs following direct duodenal instillation of single doses up to 2000 mg/kg, equivalent to 17× a daily clinical dose of 3000 mg of elemental lanthanum. Similarly, in conscious dogs given daily intravenous lanthanum chloride injections for 28 days, no adverse cardiovascular effects were observed despite achievement of extreme plasma lanthanum concentrations. The complete absence of cardiovascular effects at such high plasma concentrations suggests that circulating lanthanum ions are unavailable to bind to calcium-binding sites on cardiac calcium channels and other proteins as observed in vitro, presumably because of extensive (>99.9%) binding to plasma proteins.^23^

Electron microscopy studies indicate that lanthanum ions cannot pass through tight junctions in the blood-brain barrier;^24^,^25^ however, two studies in uremic rats reported significant levels of lanthanum in the brain, raising concerns about the potential for cognitive effects in patients.^26^,^27^ Both of these studies used high levels of lanthanum administered in a powdered diet, a method that has recently been shown to carry a high risk of tissue contamination when the brain is dissected during autopsy.^28^

Both normal and uremic rats exhibited a small increase in urine calcium excretion at high doses of lanthanum carbonate, without any significant changes in serum calcium concentration. This has been reported with other phosphate binders.^19^,^20^ Phosphate in the intestine acts as a natural calcium binder, and therefore the use of phosphate binders can lead to an increase in the amount of dietary calcium available for absorption.^21^ It is possible that the elevated urine calcium excretion is a consequence of this phenomenon. Alternatively, as lanthanum carbonate administration reduced urinary phosphate excretion almost to zero at 2000 mg/kg/day, rats may have been in negative balance at this dose due to over-efficient binding of phosphate in the intestine. Under these circumstances, phosphorus (and hence calcium) may have been mobilized from bone to maintain serum phosphorus concentrations in the physiologic range.

### Safety Pharmacology

In vitro, lanthanum ions have been widely shown to have both calcium agonist and antagonist actions, depending on the proteins or cell systems being investigated.^22^ The extent to which these effects are relevant to clinical use of lanthanum carbonate is contentious due to poor GI absorption and extensive protein binding,^23^ resulting in very low systemic levels of free lanthanum. The in vitro effects of lanthanum may be explained by its ability to substitute for calcium at binding sites on extracellular or cell-membrane-bound proteins.^22^ Intracellular processes are unaffected as lanthanum appears to be unable to enter healthy cells except by pinocytosis or phagocytosis.^22^

In our systematic evaluation of safety pharmacology, no adverse effects were observed on cardiovascular and respiratory systems in anesthetized instrumented dogs following direct duodenal instillation of single doses up to 2000 mg/kg, equivalent to 17× a daily clinical dose of 3000 mg of elemental lanthanum. Similarly, in conscious dogs given daily intravenous lanthanum chloride injections for 28 days, no adverse cardiovascular effects were observed despite achievement of extreme plasma lanthanum concentrations. The complete absence of cardiovascular effects at such high plasma concentrations suggests that circulating lanthanum ions are unavailable to bind to calcium-binding sites on cardiac calcium channels and other proteins as observed in vitro, presumably because of extensive (>99.9%) binding to plasma proteins.^23^

Electron microscopy studies indicate that lanthanum ions cannot pass through tight junctions in the blood-brain barrier;^24^,^25^ however, two studies in uremic rats reported significant levels of lanthanum in the brain, raising concerns about the potential for cognitive effects in patients.^26^,^27^ Both of these studies used high levels of lanthanum administered in a powdered diet, a method that has recently been shown to carry a high risk of tissue contamination when the brain is dissected during autopsy.^28^

The current investigations do not suggest a potential for adverse CNS pharmacology or toxicology. Of note, a neurobehavioral functional observation battery was conducted during long-term toxicity studies in mice and dogs as part of the preregistration safety evaluation of lanthanum carbonate; no adverse CNS pharmacology was related to treatment at doses of up to 1500 mg/kg/day in mice and 2000 mg/kg/day in dogs. Clinical use of lanthanum carbonate has similarly not been associated with adverse CNS effects and in a controlled study against standard treatment, no treatment-related changes in cognitive function were observed in patients undergoing dialysis given lanthanum carbonate for 2 years.^29^

As phosphate binders are administered in high doses and are not efficiently absorbed, by far the highest concentrations reside in the GI tract. Oral doses of lanthanum carbonate (<1000 mg/kg) did not induce gastric damage or exacerbate the gastric damage induced by aspirin and did not affect fecal output in rats. Intestinal transit time for a charcoal meal was not affected in rats given the same doses, although slightly reduced gastric emptying was inferred by smaller quantities of charcoal released into the intestine at 1000 mg/kg compared with controls, but not at 500 mg/kg. The main effect of lanthanum carbonate on GI physiology was an antacid action, with an approximately 2-unit increase in gastric pH at 1000 mg/kg in rats. This is analogous to the antacid action of calcium carbonate, with the carbonate ion consuming protons during its conversion to water and carbon dioxide.^30^ No effects on gastric secretion or pepsin activity occurred (using about 8× a daily clinical dose of 3000 mg elemental lanthanum), suggesting that this effect may not be relevant to therapeutic doses.

In conclusion, these in vivo studies indicate that lanthanum carbonate is one of the most potent of the currently available phosphate binders. Furthermore, in a systematic assessment of the safety pharmacology of lanthanum carbonate at supratherapeutic doses in animals, no adverse secondary pharmacological actions of concern for clinical use were identified.
